# Investigation Into Environmental Selenium and Arsenic Levels and Arseniasis Prevalence in an Arsenic-Affected Coal-Burning Area

**DOI:** 10.3389/fnut.2022.922481

**Published:** 2022-06-20

**Authors:** Ai-Mei Bai, Qian Li, Yue Li, Zhong-Xue Fan, Xiao-Qian Li, Wen-Hong Tan, Dong-Yuan Cao, Yi-Jun Kang

**Affiliations:** ^1^Key Laboratory of Trace Elements and Endemic Diseases of the National Health Commission, Shaanxi Provincial Institute For Endemic Disease Control, Xi’an, China; ^2^Key Laboratory of Shaanxi Province for Craniofacial Precision Medicine Research, Research Center of Stomatology, Xi’an Jiaotong University College of Stomatology, Xi’an, China; ^3^School of Public Health, Xi’an Jiaotong University Health Science Center, Xi’an, China

**Keywords:** arsenic, selenium, arsenic poisoning, antagonism, coal

## Abstract

This study aims to explore whether selenium (Se) concentration correlates with arseniasis in a high-arsenic coal area in the southern Shaanxi Province, China. Herein, an epidemiological investigation was conducted among 100 arsenic (As)-poisoned patients in Ziyang County, an area with high soil As and Se levels. Fifty healthy subjects were selected from areas without endemic As poisoning. The subjects in the high-As coal area were diagnosed with either normal, suspicious, mild, moderate, or severe As poisoning. Local coal, water, soil, corn, and pepper samples, as well as hair, blood, and urine samples of subjects and patients were collected and analyzed for their As and Se contents. The contents of As and Se in coal, soil, corn, pepper, and hair samples from Ziyang County were significantly higher than those in the control area. The As content of hair in Ziyang County positively correlated with As poisoning, whereas the Se content of hair and urine negatively correlated with As poisoning. The Se content in the body was negatively correlated with the degree of As poisoning, indicating that Se may accelerate the metabolism and decumulation of As and antagonize As toxicity.

## Introduction

Endemic arsenic (As) poisoning is a chronic systemic disease that seriously endangers human health (1). Epidemiological data confirm that As causes damage to the cardiovascular, digestive, immune, and peripheral nervous systems, and in serious cases may lead to tumorigenesis (2, 3). The Qinba Mountain region in the southern Shaanxi Province follows the Guizhou Province as the second area in China to sustain coal contamination by As poisoning. The As content of coal averages 197.6 mg/kg, and the affected area includes nine counties and 1.13 million people (4). The long-term burning of high-As coal for cooking, heating, and baking gradually pollutes indoor air and food items, ultimately resulting in As poisoning. Coincidentally, the Shaanxi Province is also rich in selenium (Se) (5). Both As and Se have similar chemical structures, and the latter is known for scavenging free radicals, enhancing antioxidant capacity, and protecting the liver. The As content of coal and incidence of As poisoning in this area are high, but the symptoms experienced by patients are relatively mild (6). Se and As work antagonistically in the body by competing in many biological functions (7). Se can form a complex with As to lower its toxicity to the human body (8), and several trials have shown that Se supplementation reduces As toxicity (9). This study aims to explore the relationship between Se levels in the environment and the degree of As poisoning.

## Materials and Methods

### Subjects

The study was conducted in Qinling Mountain, southern Shaanxi Province, China. The study protocols were approved by the Institutional Ethics Committee of the Shaanxi Provincial Institute for Endemic Disease Control (2018-10). All subjects gave written informed consent. Subjects over 40 years of age, who lived in the high As and Se towns of Haoping and Shuangan in Ziyang County, Shaanxi, China, were selected. All patients had lived in a high-As coal area for over 1 year and had similar lifestyles and eating habits. Healthy subjects from Baihe County, an area free from high-As coal, were selected as the control group.

The diagnosis of arseniasis was based on the history of As exposure, presence of dermal lesions (hyperkeratosis of palms and soles, and hyper- or hypo-pigmentation of the trunk), and other symptoms detailed in the technical guidelines issued by the Chinese Ministry of Health: “Diagnosis guideline for arseniasis” (WS/T211-2001). The diagnoses were divided into five grades: normal, suspicious, mild, moderate, and severe. Normal subjects did not have any abnormal skin spots but had lived in a high-As coal area for over 1 year. Suspicious arseniasis patients had only small hyperpigmentation spots, or 1–2 small keratosis spots (<3 mm), or other symptoms, such as blurred vision, diminished taste, and anorexia. Patients with mild arseniasis only had mild keratosis, scattered gray or brown spots, or scattered hypo-pigmentation. Patients with moderate arseniasis had apparent keratosis, brown-gray or darker brown hyperpigmentation spots, or more frequent hypo-pigmentation spots. Patients with severe arseniasis had keratosis appearing on the back of their hands and the insteps, palms, and soles of the feet, with densely distributed keratosis, widely spread gray-black or dense brown hyperpigmentation spots, or densely clustered hypo-pigmentation spots (10).

### Sample Collection

Coal samples were collected from five households at different locations at each survey site. The coal samples were collected using a five-point method—four points in the corners of a square and the fifth at the center (11). After crushing, the samples were blended and stored for analysis. Five tillage soil samples were collected from different locations in each survey site. Soil at a depth of 15–30 cm was collected from the tillage layer by quincunx sampling. After mixing, the soil was divided into four parts for storage purposes. Water samples from five households at different survey sites were collected in glass bottles and stored in refrigerators. Twenty local corn-and-pepper samples were collected from each survey site and stored.

All urine samples were collected and refrigerated. Hair samples were collected from the occipital skin of all subjects (11), and the collected hair was not longer than 20 mm, measured from the skin level. Venous blood samples (4.0 ml) were collected from each subject and heparin was added for anticoagulation. The serum was stored at –20°C after centrifugation.

### Sample Pretreatment

#### Coal and Soil

Coal and soil samples were dried at 60°C in a thermostatic dryer and then ground to pass through a 100-mesh sieve. Each sample (0.50 g) was added to a 150-ml conical flask, and 10 ml of a mixture of nitric acid and perchloric acid was added (4:1 by volume). The flask was left overnight. The completely digested samples were then heated on an electric plate until the sample solution became colorless and transparent.

#### Hair

The hair sample was cut into small fragments and put in a 100-ml beaker each. Each sample fragment was washed repeatedly with detergent and rinsed three times with pure water before being dried at 60°C in a thermostatic dryer. Each sample fragment (0.50 g) was placed in a 150-ml conical flask, and 10 ml of the acid mixture was added. The flasks were left overnight. The samples were then digested on a sand-bath heating plate until the solution became clear.

#### Serum

Each serum sample (2 ml) was accurately pipetted into a 150-ml conical flask, and 10 ml of the acid mix was added. The next day, the sample was heated and digested on a sand-bath heating plate until the solution volume was reduced to 2 ml. The solution became bright green and transparent and was then cooled to room temperature. The sample solution was transferred to a 25-ml volumetric flask with 10% hydrochloric acid, followed by addition of 50% hydrochloric acid 2.5 ml and 5% thiourea ascorbic acid 5 ml, brought to the volume with 10% hydrochloric acid, and shaken well. Testing was then performed on the machine 30 min later.

#### Corn and Pepper

Impurities were removed from the corn-and-pepper samples before they were dried in a thermostatic oven at 60°C. The sample was pulverized and passed through a 40-mesh sieve. A corn or pepper sample (1.0 g) was placed in a 150-ml conical flask and 10 ml of mixed acid was added. The flask was covered with a glass plate and left overnight in a fume hood. The next day, the solution was heated and digested on an electric heating plate until clear and colorless and was then cooled to room temperature. The sample solution was transferred into a volumetric flask with 5 ml of 1% potassium ferricyanide and 5 ml of 5% thiourea ascorbic acid, and the volume was adjusted to 25 ml with 10% hydrochloric acid.

#### Urine

Urine (1 ml) was pipetted into a digestion tube, to which 0.25 ml of 50% nitric acid and 0.5 ml of 30% hydrogen peroxide were added. The next day, the solution was slowly heated and digested on the sand-bath heating plate until it was colorless and transparent. The solution volume was reduced to 0.2 ml and cooled to room temperature. Hydrochloric acid (1 ml, 50%) and thiourea ascorbic acid (2 ml, 5%) were added, and the volume was adjusted to 10 ml with pure water.

### Arsenic and Selenium Contents

After pretreatment, the As and Se contents were determined using an AFS-930 double-channel atomic fluorescence spectrometer (Jitian Instrument, Beijing, China).

### Statistical Analysis

Statistical analysis was performed using SPSS statistical software (version 17.0). Data with a normal distribution were presented as the mean ± SEM. Data that did not satisfy the conditions for normal distribution and homogeneity of variance were represented by the median (M) and quartile range (QR). The As and Se levels in hair, blood, and urine samples were compared using the non-parametric rank-sum test. Correlation analysis was performed using the two-variate Spearman correlation method. A default confidence level of 95% was used. A value of *p* < 0.05 was considered significant.

## Results

### Screening for Subjects

In this study, an epidemiological investigation was conducted on a population over 18 years of age, living in the towns of Haoping and Shuangan in Ziyang County and Chengguan in Baihe County. A total of 232 persons were diagnosed with arseniasis out of the 2,299 subjects from Haoping Town, giving a prevalence rate of 10.09%. Fifty-nine persons out of the 1,233 subjects from Shuangan Town were diagnosed with arseniasis (prevalence rate of 4.79%), and no arseniasis was diagnosed among the 1,220 patients in Baihe County. According to the results of a 5-year monitoring program tracking arseniasis cases caused by coal-burning pollution in the Shaanxi Province, the detection rate of arseniasis in people over 40 years of age gradually increased. Therefore, 100 patients over 40 years of age in Haoping and Shuangan towns were randomly selected as subjects.

### Arsenic Contents in Coal, Soil, Water, Pepper, and Corn

**FIGURE 1:**
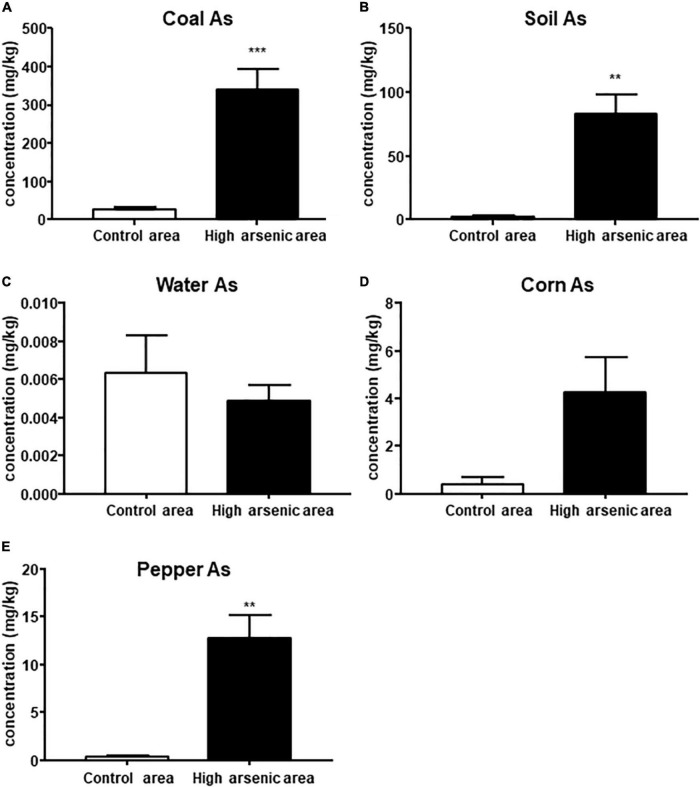
Arsenic content of coal, soil, water, corn, and pepper in Ziyang County and the control area. Data are represented by mean ± SEM. The comparison was performed by *t*-test. **, *** *p* < 0.01 and 0.001, respectively.

In Ziyang County, the As content of coal was estimated to be 341.60 ± 193.83 mg/kg, which was significantly higher than that specified in the Definition and Division Standard for Endemic Arsenism (WS277-2007: 40 mg/kg). The As content of the soil in this area was 83.07 ± 53.14 mg/kg, significantly higher than that specified in the hygiene standard for As in soil (GB8915-1988: 15 mg/kg). The As contents of corn and pepper were 4.24 ± 6.77 mg/kg and 12.80 ± 23.70 mg/kg, respectively, both significantly higher than those defined in the National Standard for Maximum Levels of Contaminants in Food (GB2762-2005: 0.2 mg/kg for coarse cereals, and 0.05 mg/kg for vegetables). By contrast, the As content of water was lower than the National Hygienic Standard of Living Water (GB5749-2006: 0.01 mg/l). Compared to the control area, the As content in coal, soil, and pepper from Ziyang County was significantly higher (*p* = 0.0005, 0.0020, and 0.0018, respectively; Figure 1). However, there were no significant differences in the As content of water and corn (*p* = 0.4248 and 0.1240, respectively; Figure 1).

### Selenium Contents in Coal, Soil, Water, Pepper, and Corn

**FIGURE 2:**
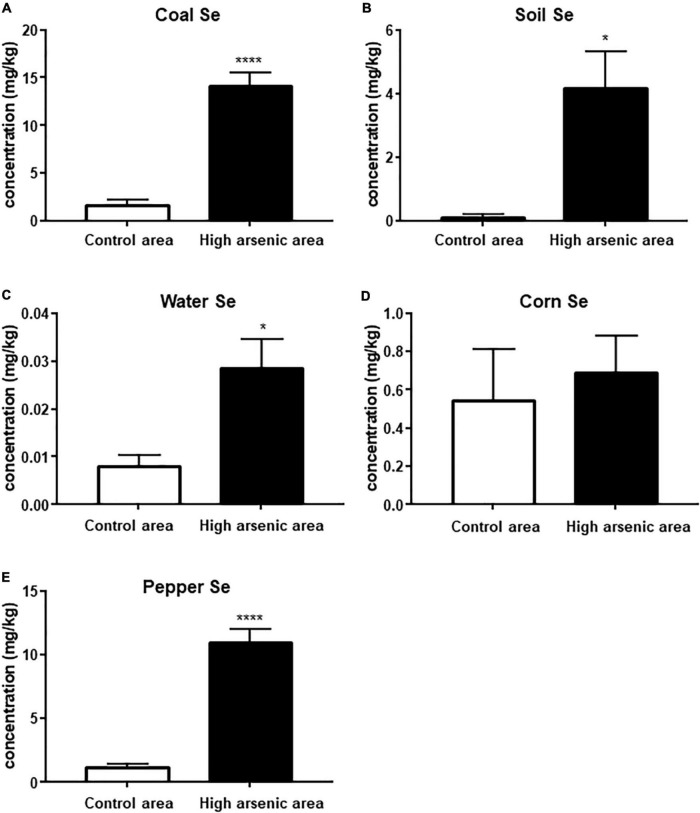
Selenium content of coal, soil, water, corn, and pepper in Ziyang County and the control area. Data are represented by mean ± SEM. The comparison was performed by *t*-test. *, **** *p* < 0.05 and 0.0001, respectively.

The coal Se content in the high-As area was 14.10 ± 4.95 mg/kg, greater than both the National Selenium Standard (6.22 mg/kg) and the Worldwide Selenium Standard (3.0 mg/kg). The water-soluble Se content of the soil was 2.410 ± 3.970 mg/kg, higher than the average water-soluble Se content of the soil in China (0.010 mg/kg). The Se content of water (0.028 ± 0.019 mg/l) was also higher than the average value in China (0.01 mg/l). The Se contents in corn and pepper were significantly higher than those defined in the National Standard for Maximum Levels of Contaminants in Food (0.3 and 0.1 mg/kg, respectively). Compared to the control area, the Se levels in coal, soil, water, and pepper in the high-As area were significantly higher (*p* < 0.0001, *p* = 0.0258, *p* = 0.0396, and *p* < 0.0001, respectively; Figure 2). However, there was no significant difference in the Se content of corn (*p* = 0.6743; Figure 2).

### Arsenic Content in Hair, Serum, and Urine

**FIGURE 3:**
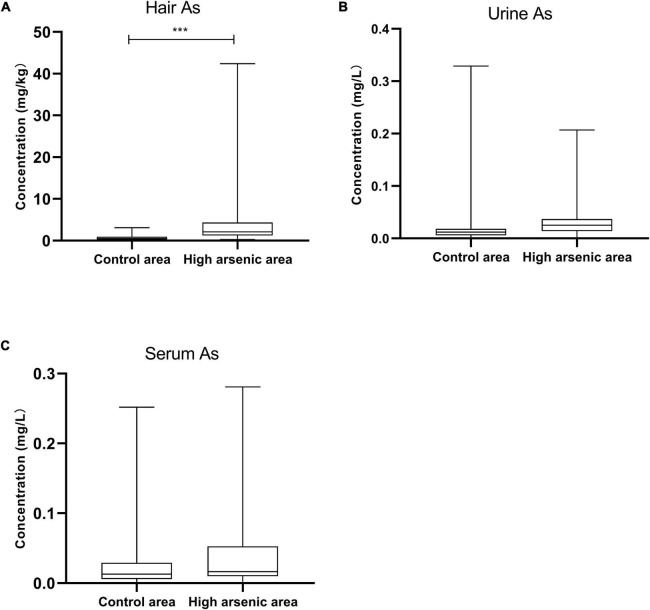
Arsenic content of hair, urine, and serum in Ziyang County and the control area. Data are represented by median (M) and quartile range (QR). The comparison was performed by the non-parametric rank-sum test. *** *p* < 0.001.

The As content of the hair samples from Ziyang County was higher than that of the control area samples (*p* = 0.0002). However, there were no significant differences in the As content of the urine and serum samples (*p* = 0.8145 and 0.1155, respectively; Figure 3).

### Selenium Content in Hair, Serum, and Urine

**FIGURE 4:**
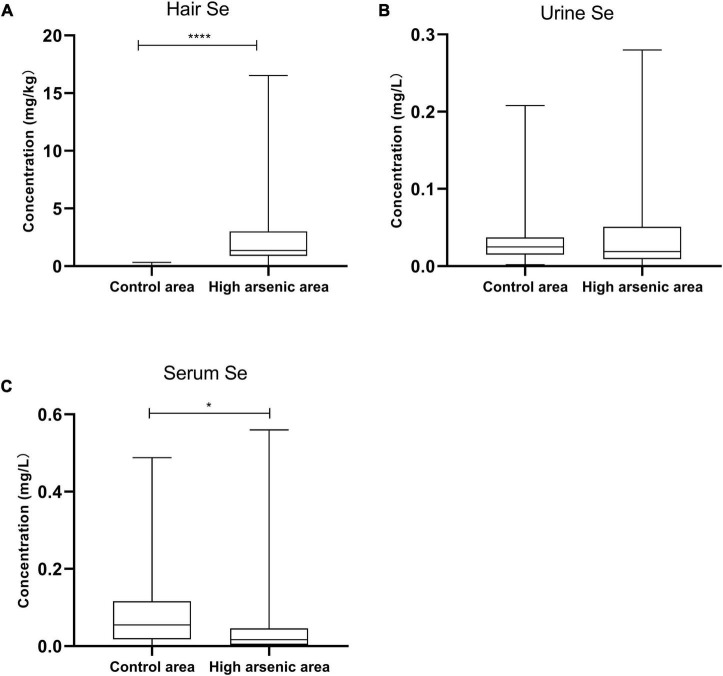
Selenium content of hair, urine, and serum in Ziyang County and the control area. Data are represented by median (M) and quartile range (QR). The comparison was performed by the non-parametric rank-sum test. *, **** *p* < 0.05 and 0.0001, respectively.

The Se content of hair and serum samples in the high-As area was significantly higher than those in the control area (*p* < 0.0001 and *p* = 0.0293, respectively). However, there was no significant difference in the Se content of urine (*p* = 0.6415; Figure 4).

### Clinical Classification of Arsenic Poisoning in Patients

The 100 patients investigated were distributed between the diagnosis categories as follows: severe (4%), moderate (20%), mild (48%), and suspicious (28%) (Table 1). There was a positive correlation between the degree of As poisoning and the As content of hair samples in the high-As area (*r* = 0.862, *p* < 0.01). However, there was a negative correlation between the Se content of hair and urine, and the degree of As poisoning in the high-As area (*r* = –0.349 and *r* = –0.344, respectively; *p* < 0.01).

**TABLE 1:** Clinical classification of arsenic poisoning in patients.

Site	Case number		Clinical classification [case, constituent ratio (%)]			
		Normal	Suspicious	Mild	Moderate	Severe
High arsenic area	100	0 (0)	28 (28)	48 (48)	20 (20)	4 (4)
Control area	50	50 (100)	0 (0)	0 (0)	0 (0)	0 (0)

## Discussion

Chronic As poisoning damages the skin, liver, kidneys, lungs, and peripheral and central nervous systems. After As enters the body, methylation and secondary methylation lead to modified forms of As with different valence states (12, 13). As metabolites are cytotoxic and may increase the risk of human squamous cell carcinoma (14, 15). Se, by contrast, is an important trace element in the human body and is crucial for mitigating oxidative damage. It can scavenge free radicals, increase antioxidant capacity, and protect the liver. The chemical structures of As and Se are similar, with As being an oxidant and Se an antioxidant. Se suppresses the toxicity of As through methylation inhibition (3), as the methylation of As is one of its important metabolic pathways. As and Se are absorbed in the body through the respiratory tract, digestive tract, as well as skin and mucous membrane. Therefore, the contents of As and Se in the environment directly affect the levels of As and Se in the body, impacting the metabolism and physiological functions of these elements.

### Distribution of Arsenic and Selenium in the External Environment

The As content of water in Ziyang County was below the standard value, suggesting that this was not affected by As poisoning. However, the excessive As content measured in the coal samples is the characteristic of this area. When contaminated coal burns, As diffuses into the air in the form of gaseous As_2_O_3_ and As-enriched fly ash. When residents use high-As coal for cooking, heating, and baking, the As content increases in the indoor air, grain, peppers, and other food items as a result of secondary pollution. The residents then pile or scatter the coal cinder on the ground, thus contaminating the soil (coal cinder As content 73.97–218.47 mg/kg; average 133.10 + 37.58 mg/kg) (16). In addition, local coal mines are widely distributed and shallowly located. Therefore, the land is contaminated by As from both raw coal and coal cinders. This study found that the average As content of coal in this area was more than eight times that specified in the Definition and Division Standard for Endemic Arsenism (WS277-2007: 40 mg/kg) and more than three times the Chinese coal industry standard (MT/T803-1999: 100 mg/kg). The average As content of soil was more than five times that of the Hygiene Standard for As in Soil (GB8915-1988: 15 mg/kg), and the average As contents of corn and pepper (12.80 ± 23.70 mg/kg) were higher than those defined in the National Standard for Maximum Levels of Contaminants in Food (GB2762-2005: 0.2 mg/kg for coarse cereals, 0.05 mg/kg for vegetables). Ziyang County is known to have the second-highest Se content in China (17). The average Se content of the coal samples from this area is higher than both the Se standard of China (6.22 mg/kg) and the worldwide standard (3.0 mg/kg). The average amount of water-soluble Se in the soil and the Se content in the drinking water were significantly higher than those in other areas. Furthermore, the average amounts of Se in corn and pepper were significantly higher than those defined in the national standard (0.3 and 0.1 mg/kg, respectively).

### Levels of Arsenic and Selenium in Body

Given the excessive levels of As in the external environment, the hair samples from Ziyang County contained substantially more As than those from the control area. Additionally, the levels of Se in the hair, serum, and urine samples from Ziyang County were higher than the corresponding standards (0.8 mg/kg, 0.1–0.34 mg/l, and 0.026–0.012 mg/l, respectively) but lower than the toxic values (3.70 mg/kg, 0.44 mg/l, and 0.33 mg/l, respectively). Thus, the levels of Se in the hair, urine, and serum samples were high but not toxic, related to the high levels of Se in the environment. Hair is the most readily obtainable biological specimen for determining As exposure and has thus been used to monitor long-term As pollution (17, 18). An As level beyond 3 mg/kg in hair samples is considered abnormally high, whereas values below 1 mg/kg indicate low As exposure (17, 18). As the levels of As vary significantly across biological specimens, the specimens should be collected from a range of different individuals (17).

### Clinical Grading of Arsenic Poisoning Patients

The arseniasis cases were identified by symptoms of dermal lesions (hyperkeratosis of palms and soles and hyper- or hypo-pigmentation of the trunk), according to the technical guidelines issued by the Chinese Ministry of Health: “Diagnosis Guideline for Arseniasis, WS/T211-01” (10, 19–21). Most patients in Ziyang County were found to have symptoms in the mild and suspicious diagnosis categories (defined previously) with no organ damage, no symptoms of fatal illness, and no signs of cancer. Spots of hyper- and hypo-pigmentation on the trunk were common, and some patients had skin keratosis. The situation in this area thus appeared better than that in the Guizhou Province of China, where the symptoms of As poisoning from burning high-As coal are more serious; skin keratosis is common, and the mortality rate is high (22). This may be due to low Se levels in the soil and an insufficient dietary intake of Se (22). The results of this study show that the degree of As poisoning correlated positively with the As content of hair and negatively with the Se content of hair and urine samples. This suggests that the higher the Se content in the environment and food, the higher the Se content in the body, which may antagonize excessive As and thus serve as an important factor causing the mild degree of As poisoning in the southern Shaanxi Province.

### Arsenic’s Metabolism and Selenium’s Function

Inorganic As can be metabolized *in vivo* to produce different forms of arsenide, with different toxicities and target organs. Se is an essential micronutrient for the human body, and it maintains immune function and enhances metabolism and detoxification. Se deficiency may exacerbate the clinical symptoms of As poisoning (23), while appropriate doses of Se can antagonize the effects of As on antioxidant inhibition (24), significantly improving the function of the body’s immune system (25). A recent animal study showed that a high-Se diet increased urinary and fecal As excretion, reduced renal As residues, enhanced antioxidant levels, and reversed As-induced immune suppression (26). This study found that although people living in an area with high As pollution were exposed to high levels of As, the incidence of As poisoning was relatively low. This may be attributed to the high levels of Se in the external environment and high levels of Se in the body. Therefore, it seems that As poisoning may be prevented by eliminating the source of As, reducing the entry of As into the body, increasing the internal Se levels, and promoting the excretion of As from the body. The elimination of As exposure is the key to preventing these adverse health effects (27). It is suggested that the prevention and control measures put forward by the Chinese government be implemented to cut off the source of As, reduce the entry of As into the human body, develop As-repellent drugs, and promote the excretion of As from the body. Furthermore, appropriate supplementation of Se can be used to increase the internal Se levels of residents in As poisoning areas, aiding the prevention or treatment of endemic As poisoning.

## Data Availability Statement

The raw data supporting the conclusions of this article will be made available by the authors, without undue reservation.

## Ethics Statement

The studies involving human participants were reviewed and approved by the Institutional Ethics Committee of Shaanxi Provincial Institute For Endemic Disease Control. The patients/participants provided their written informed consent to participate in this study.

## Author Contributions

A-MB and Y-JK conceived and designed the study. A-MB, QL, YL, Z-XF, X-QL, and W-HT collected the data and performed the data analyses. A-MB got the funding. A-MB, QL, D-YC, and Y-JK wrote the initial draft of the manuscript. W-HT, X-QL, YL, and Z-XF revised the manuscript. All authors have contributed to the manuscript and approved the submitted version.

## Conflict of Interest

The authors declare that the research was conducted in the absence of any commercial or financial relationships that could be construed as a potential conflict of interest.

## Publisher’s Note

All claims expressed in this article are solely those of the authors and do not necessarily represent those of their affiliated organizations, or those of the publisher, the editors and the reviewers. Any product that may be evaluated in this article, or claim that may be made by its manufacturer, is not guaranteed or endorsed by the publisher.
